# Aging is associated with glial senescence in the brainstem - implications for age-related sympathetic overactivity

**DOI:** 10.18632/aging.203111

**Published:** 2021-05-26

**Authors:** Priya Balasubramanian, Lyndee Branen, Mahesh Kumar Sivasubramanian, Raisa Monteiro, Madhan Subramanian

**Affiliations:** 1 Oklahoma Center for Geroscience and Healthy Brain Aging, The University of Oklahoma Health Sciences Center, Oklahoma City, OK 73104, USA; 2 Department of Physiological Sciences, College of Veterinary Medicine, Oklahoma State University, Stillwater, OK 74078, USA

**Keywords:** senescence, brainstem, aging, glial cells, sympathetic nervous system

## Abstract

Accumulating evidence suggests that the sympathetic nervous system (SNS) overactivity plays a crucial role in age-related increase in the risk for cardiovascular diseases such as hypertension, myocardial infarction, stroke and heart diseases. Previous studies indicate that neuroinflammation in key brainstem regions that regulate sympathetic outflow plays a pathogenic role in aging-mediated sympathoexcitation. However, the molecular mechanisms underlying this phenomenon are not clear. While senescent cells and their secretory phenotype (SASP) have been implicated in the pathogenesis of several age-related diseases, their role in age-related neuroinflammation in the brainstem and SNS overactivity has not been investigated. To test this, we isolated brainstems from young (2-4 months) and aged (24 months) male C57BL/6J mice and assessed senescence using a combination of RNA-*in situ* hybridization, PCR analysis, multiplex assay and SA-β gal staining. Our results show significant increases in p16^Ink4a^ expression, increased activity of SA-β gal and increases in SASP levels in the aged brainstem, suggesting age-induced senescence in the brainstem. Further, analysis of senescence markers in glial cells enriched fraction from fresh brainstem samples demonstrated that glial cells are more susceptible to senesce with age in the brainstem. In conclusion, our study suggests that aging induces glial senescence in the brainstem which likely causes inflammation and SNS overactivity.

## INTRODUCTION

In the aging population (>65 years old), cardiovascular diseases rank as the leading cause of mortality and contribute to 40% of all deaths in the US [1]. A recent report from the American Heart Association (2016) states that more than 80% of persons aged 65 and older have some form of cardiovascular disease (CVD) costing $300 billion annually in health care costs [2]. The projections predict that by 2035, across all conditions, total CVD costs will more than triple among those aged 80+ and more than double among those aged 65-79 which will create a substantial burden on taxpayers. With the rising aging population across the globe, the identification of novel interventions to treat CVDs is a health care imperative.

Age-related pathological alterations of the heart and the vasculature likely contribute to the higher prevalence of cardiovascular diseases in the elderly. One of the centrally mediated mechanisms that is implicated in the pathogenesis of CVDs with aging is sympathetic nervous system (SNS) dysregulation [3–5]. Multiple lines of evidence demonstrate overactive SNS with advanced age. Direct nerve recordings show an increase in burst discharge rate of muscle sympathetic nerve activity (SNA) in older men when compared to young men [6, 7] and increased muscle SNA is an important predictor of mortality in chronic heart failure patients [8]. Furthermore, studies report increases in resting SNA [9] with higher total norepinephrine (NE) spillover, hepatomesenteric and cardiac NE spillover in older individuals compared to young adults [4, 10]. Aging is also associated with decreased sensitivity of arterial baroreceptor reflex in humans [6]. Also, lower heart rate variability reported in older individuals indicates decreased parasympathetic input to the heart with aging [11]. Chronic increases in SNA to heart and blood vessels are clinically manifested as increases in systolic blood pressure, diastolic dysfunction and increases in ventricular and aortic wall thickness, which comprises the classical hallmarks of cardiovascular aging in humans. However, the neural mechanisms behind the increase in SNA [12] observed with aging are not clear.

Cellular senescence is a state of irreversible growth arrest that occurs in proliferating cells in response to cellular damage or sub-lethal stress induced by telomere shortening, oxidative stress, oncogene activation, DNA damage or mitochondrial dysfunction [13]. Senescent cells are heterogeneous [14, 15] and their identification usually, involves a combination of several biochemical markers, such as increased expression of cell cycle inhibitors (p16^Ink4a^, p21^Cip1^), increased senescence-associated β-galactosidase activity (SA-βgal), reduced LaminB1, and other cytoskeletal changes including heterochromatin foci and DNA damage foci [16]. Further, senescent cells affect neighboring cells through the acquisition of senescence-associated secretory phenotype (SASP) and secretion of pro-inflammatory cytokines, chemokines, growth factors and proteases. Senescent cells have physiological roles in tumor suppression and wound healing [16, 17]. However, increase in the accumulation of these cells with aging have been shown to promote tissue deterioration and drive the progression of age-related diseases [18–22]. Emerging studies have also demonstrated that senescent cells, mostly glial cells in the hippocampus and cortex, drive neurodegenerative diseases like Alzheimer’s (AD) and Parkinson’s disease during aging [23–25]. Taking into consideration that age-related SNS dysregulation is mediated by neurogenic mechanisms [3, 4, 26, 27], the possibility that senescence in the CNS cells may be a contributing factor should not be overlooked. Particularly, senescence-mediated inflammation through the SASP is relevant as neuroinflammation in the brainstem nuclei like the rostral ventrolateral medulla (RVLM, a key region that regulates basal and reflex control of sympathetic nerve activity) contributes to sympathoexcitation in several models of essential hypertension and heart failure [28–30]. Hence in this study, we sought to investigate for the first time whether the age-related increase in SNS activity is associated with cellular senescence in the brainstem. Further, we also investigated what type of cells are more prone to senesce with age in the brainstem.

## RESULTS

### Aging increases whole-body SNA

**Figure 1:**
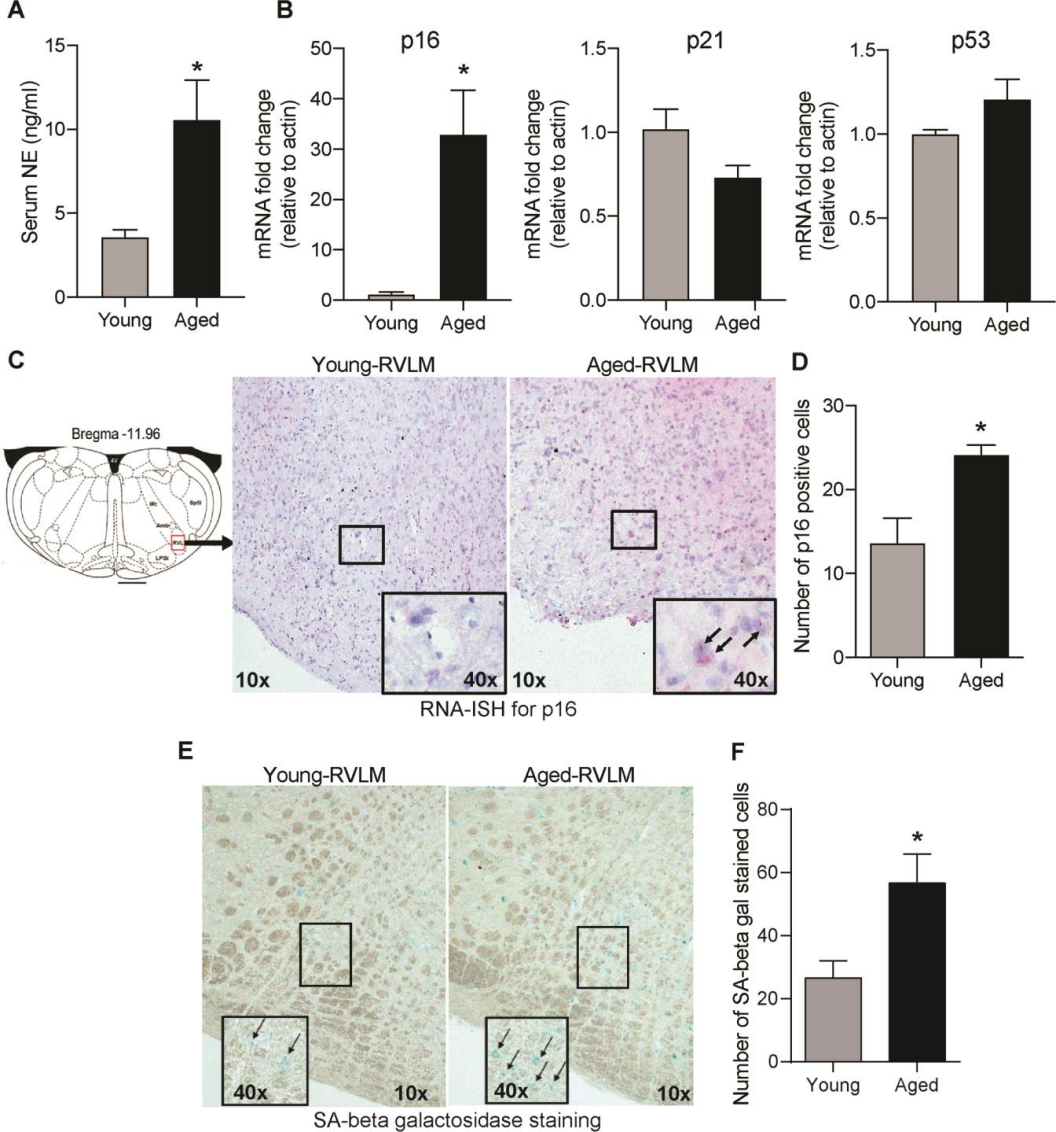
**Age-induced changes in serum NE and senescence markers in the brainstem.** (**A**) Changes in serum NE levels in young and aged animals measured using a commercial ELISA kit (mean±SE, n=4-6/group). (**B**) Real-time PCR analysis of gene expression levels of senescence markers p16, p21 and p53 (n=4/group) (**C**) Representative images of RNA-ISH showing p16-positive cells in the RVLM of the brainstem in young and aged animals. (**D**) Semi-quantitative analysis measuring the number of p16-positive cells in the RVLM by RNA-ISH. (**E**) Representative images of SA-β gal staining in the brainstem and (**F**) Quantification of cells positive for SA-β gal staining in the RVLM of the brainstem. *denotes a significant difference (p < 0.05) from young animals.

To confirm previous findings on age-related sympathetic overactivity, we measured circulating norepinephrine (NE) levels as a surrogate marker for whole-body SNA in young and aged animals. Aged animals had higher serum NE levels than young controls (Figure 1A), indicating overactive SNS with aging.

### Aging induces senescence in the brainstem

Senescent cell accumulation with aging has been documented in several peripheral tissues and also in brain regions like the hippocampus and cortex in neurodegenerative diseases like Alzheimer’s disease, Parkinson’s disease and multiple sclerosis [23, 24, 31]. In this study, we wanted to investigate whether aging increased the expression of senescence markers in the brainstem, the region that regulates SNA. First, we measured the gene expression levels of classical senescence markers like p16, p21 and p53 that are involved in cell cycle regulation. We observed a 32-fold increase in p16 mRNA expression in the aged brainstem compared to young controls (Figure 1B). However, there were no changes in p21 or p53 expression in the brainstem with aging (Figure 1B). To confirm if senescence occurs in the rostral ventral lateral medulla (RVLM), a key region in the brainstem involved in tonic and reflex mediated control of SNA, we performed RNA-ISH to measure p16 mRNA expression specifically in the RVLM using formalin-fixed brainstem sections. Confirming our real-time PCR data, we observed an increase in the number of cells positive for p16 mRNA in the RVLM of aged animals when compared to young controls (Figure 1C, 1D). Further, SA-β gal staining showed a significantly higher number of positively stained cells in the RVLM of aged animals compared to young controls (Figure 1E, 1F), suggestive of age-induced senescence in the RVLM of the brainstem.

### Aging increases nuclear NF-κB activity and SASP-mediated inflammation in the brainstem

**Figure 2:**
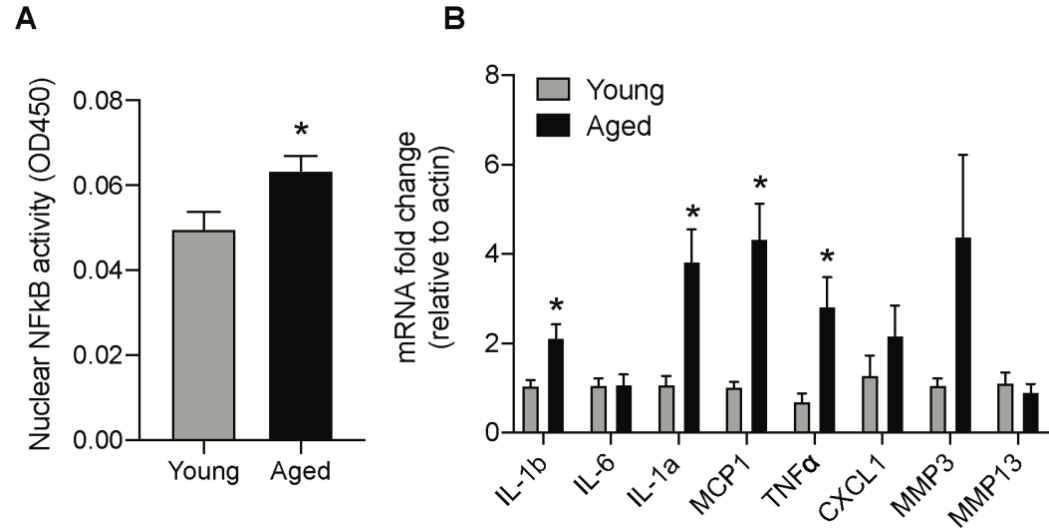
**Age-induced changes in NF-κB activity and mRNA levels of SASP factors in the brainstem.** (**A**) NF-κB DNA-binding capacity measured by ELISA in nuclear protein extracted from the brainstem of young and aged animals. (**B**) Gene expression levels of SASP factors in the brainstem measured by real-time PCR analysis. Data are expressed as mean±SE, n=4-5/group. *denotes a significant difference (p < 0.05) from young animals.

One of the major mechanisms senescent cells utilize to affect neighboring cells is through SASP, which leads to the inflammatory milieu and tissue dyshomeostasis. Nuclear factor-κB (NF-κB) subunit p65 is a master transcription factor that has been shown to accumulate on the chromatin of senescent cells and regulate the expression of SASP genes [32]. Hence, we investigated the ability of nuclear NF-κB subunit p65 to bind with DNA consensus sequence, as it provides insights about NF-κB translocation and subsequent transcriptional activity to induce SASP related genes. Nuclear protein extracted from the brainstem of aged and young animals was used to assess NF-κB subunit p65 DNA binding activity using the Trans-AM assay. As shown in Figure 2A, the nuclear NF-κB subunit p65 binding activity was significantly enhanced in the brainstem of aged animals when compared with young controls.

Next, we investigated the protein and gene expression levels of major SASP factors in the aged brainstem. The gene expression levels of IL-1b, IL-1a, MCP1, and TNFα were significantly higher while CXCL1 and MMP3 showed a trend towards an increase in the aged brainstem (Figure 2B). Also, we used the mouse 32-plex cytokine/chemokine assay to measure several different cytokines and chemokines from the brainstem protein extracts. Some of the key SASP components that were significantly upregulated at the protein level in the aged brainstem include Eotaxin, G-CSF, IL-3, IP-10, KC, LIF, MCP1, M-CSF, MIP-1b and RANTES (Table 1).

**Table 1:** Cytokine and chemokine levels measured in brainstem protein lysates using a multiplex assay in young (4 months) and aged (24 months) C57BL/6J mice.

**Cytokines (pg/μg protein)**	**Young (mean±SE)**	**Aged (mean±SE)**	**p-value**
Eotaxin	0.292±0.01	0.557±0.04	**0.0003**
G-CSF	0.515±0.04	0.781±0.06	**0.007**
GM-CSF	1.40±0.09	1.324±0.07	0.536
IFN-gamma	0.282±0.03	0.275±0.02	0.86
IL-1a	2.811±0.21	3.09±0.20	0.378
IL-1b	0.974±0.10	1.059±0.08	0.532
IL-2	0.704±0.06	0.661±0.05	0.583
IL-3	0.109±0.01	0.144±0.006	**0.02**
IL-5	0.096±0.01	0.097±0.01	0.953
IL-6	0.157±0.02	0.188±0.01	0.147
IL-7	0.242±0.02	0.230±0.02	0.705
IL-9	7.292±1.0	6.734±0.35	0.561
IL-10	3.238±0.30	3.312±0.25	0.854
IL-12 (P40)	0.578±0.05	0.615±0.06	0.66
IL-12 (P70)	1.576±0.15	1.525±0.11	0.787
IL-13	2.686±0.29	3.186±0.35	0.319
IL-15	3.53±0.25	3.331±0.21	0.552
IL-17	0.073±0.006	0.076±0.004	0.75
IP-10	0.827±0.04	3.293±0.42	**0.0006**
KC	0.819±0.06	1.231±0.09	**0.007**
LIF	0.054±0.003	0.091±0.006	**0.001**
LIX	1.957±0.20	2.199±0.22	0.464
MCP1	1.036±0.09	1.515±0.06	**0.0009**
M-CSF	2.127±0.22	2.915±0.16	**0.01**
MIG	0.389±0.04	0.588±0.14	0.273
MIP-1a	1.332±.012	1.595±0.1	0.121
MIP-1b	1.492±0.12	1.864±0.11	**0.04**
MIP-2	2.891±0.18	3.059±0.15	0.497
RANTES	0.780±0.06	1.669±0.07	**<0.005**
TNFa	0.207±0.02	0.226±0.02	0.46
VEGF	0.104±0.01	0.092±0.01	0.524

Significant changes are highlighted in bold text (p < 0.05).

### Aging increases senescence in the glial cells of the brainstem

**Figure 3:**
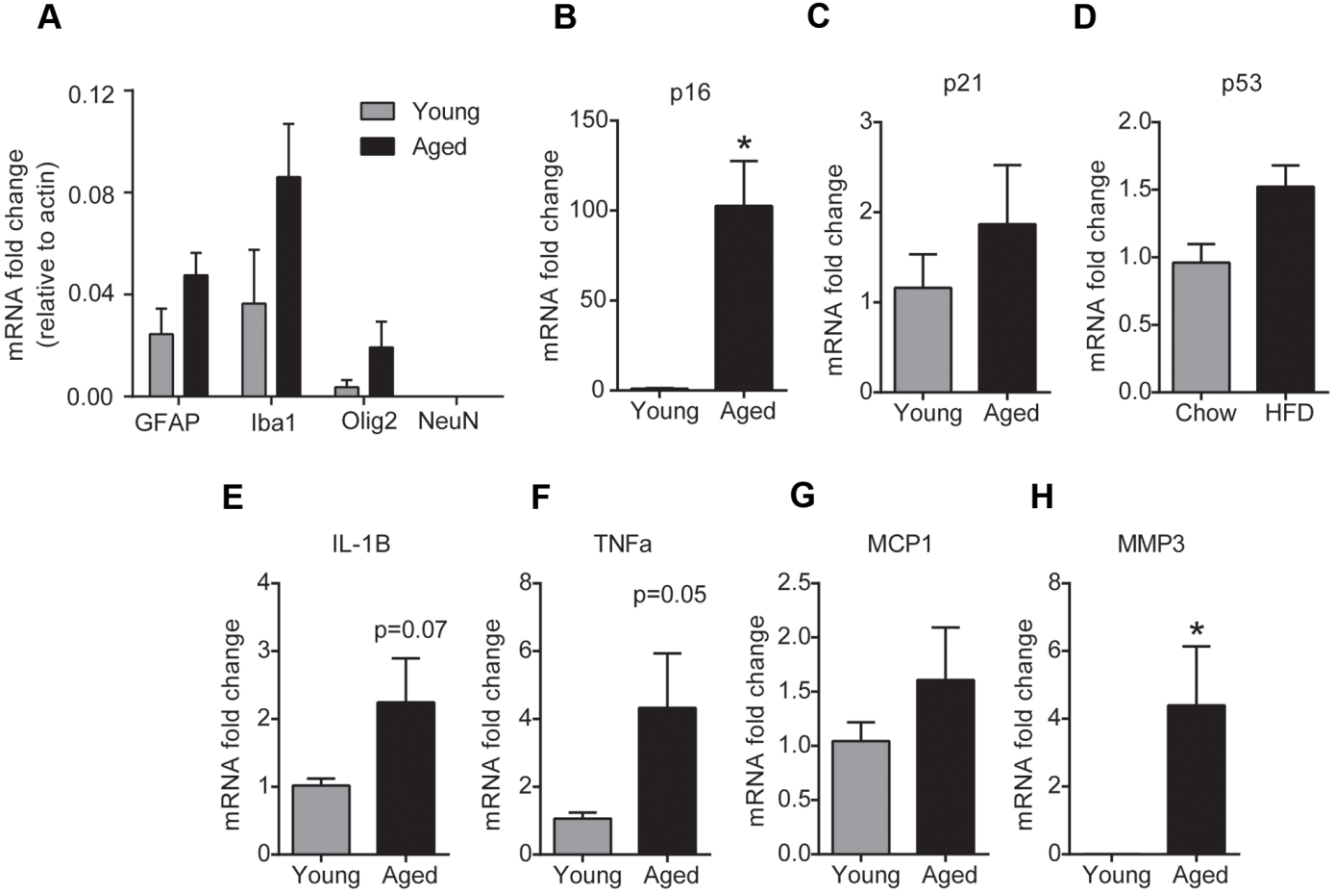
**Aging induces glial senescence in the brainstem.** (**A**) Purity of glial cell-enriched fraction assessed by real-time PCR analysis. (**B**–**H**) Gene expression analysis of senescence markers in the glial cell-enriched fraction from young and aged brainstem. Data are expressed as mean±SE, n=4-5/group. *denotes a significant difference (p < 0.05) from young animals.

To investigate the phenotype of cells that undergo senescence in the brainstem with aging, we performed gene expression analysis for senescence and SASP markers in glial cells and neurons isolated from fresh brainstem tissue using magnetic beads. To confirm enrichment for glial cells, we first analyzed the expression of GFAP (astrocyte marker), Iba1 (microglial marker), Olig2 (oligodendrocyte marker) and NeuN (neuronal marker) in the isolated cell fractions. As expected, the glial fraction was enriched for cells that had higher GFAP, Iba1 and Olig2 expression and undetectable NeuN expression indicating that there is no neuronal contamination in the glial fraction (Figure 3A). PCR analysis in the glia enriched cell population revealed that glial cells from the aged brainstem had a ~90 fold increase in the mRNA expression of p16 compared to glial cells from the young brainstem (Figure 3B). In line with the tissue expression levels, we did not observe any changes in p21 or p53 gene expression levels in the glial cells of the brainstem with aging. Also, we observed a significant increase in MMP3 levels and a trend toward an increase in TNFα and IL-1β levels in the glial cells of the aged brainstem (Figure 3E–3H). Unfortunately, our preparation did not yield viable neurons for RNA extraction and hence could not perform a similar analysis for senescence markers in brainstem neurons. Although our results do not exclude the possibility of neuronal senescence, we have confirmed the induction of p16-mediated senescence in the glial cells of the brainstem with aging.

## DISCUSSION

Age-related sympathoexcitation has a continuing interest in the field, as SNS pathophysiology is a causal factor in the increasing incidence of a range of cardiovascular diseases including systolic hypertension, cardiac failure and ventricular arrhythmias with age [27]. It is well established that the brainstem nuclei such as RVLM and Nucleus tractus solitarius (NTS) play a critical role in tonic and reflex control of SNA to peripheral tissues [33]. Pathological changes such as oxidative stress, inflammation and endoplasmic reticulum (ER) stress at the level of the brainstem have been demonstrated to contribute to sympathoexcitation in essential hypertension, heart failure and obesity [28–30]. However, very little is known about the central molecular mechanisms regulating sympathoexcitation in aging.

Accumulation of senescent cells with aging has been well established in peripheral tissues [34, 35]. Emerging studies have reported a similar pattern in the brain where an age-associated increase in senescent cells in regions like the hippocampus and cortex has been implicated in the pathogenesis of neurodegenerative diseases like tau-dependent pathologies and Parkinson’s disease [23, 24, 36]. Currently, there is no unique marker to detect senescent cells in tissues. Hence, we used a combination of several markers including expression of cell cycle inhibitors (p16 and p21), lysosomal activity (such as SA-β-Gal), and SASP secretion to identify senescent cells in the brainstem with aging as per the recommendations set forth by the International Cell Senescence Association (ICSA) [37]. Our results demonstrate for the first time that aging was associated with the accumulation of senescent cells in the brainstem, especially in the RVLM, as marked by increases in p16 expression, SA β-gal activity, increased nuclear NF-κB activity and SASP-mediated inflammation. In our study, we did not detect age-associated changes in other senescence markers like p21 or p53 in the brainstem. This is consistent with other studies which also show age- and tissue-specific variable expression of p16 and p21 [34, 35]. While advanced age increased both p16 and p21 in some tissues (liver, kidneys, pancreas, epidermis), that was not the case in other tissues like the cortex and heart where p16 is preferentially increased [34, 35]. These results suggest that there is a distinct pattern in the expression of senescence markers in various tissues, further highlighting the complexity of the senescence program and the need to establish tissue-specific biomarkers for senescence with aging.

Another important finding from our study is that glial cells undergo p16-dependent senescence in the brainstem, which has the potential to exert negative effects on pre-sympathetic neurons of the brainstem and result in sympathoexcitation. It should be mentioned that our experimental strategy does not distinguish what type of glial cells undergo senescence in the brainstem during normal aging as our preparation enriches astrocytes, microglia and oligodendrocytes. The cause of senescence induction in glial cells during aging could be multifactorial. Replicative senescence due to telomere shortening is one of the primary causes of senescence with aging, particularly in the peripheral tissues where the cell turnover rate is high [38]. While neurons are post-mitotic and do not proliferate, there is considerable evidence to show that most glial cells can proliferate and hence are capable of undergoing replicative senescence [24, 25, 39]. Bhat et al showed evidence for replicative senescence in astrocytes where the isolated primary astrocytes from postmortem AD patients underwent growth arrest in late passages when compared to early passages [40]. Similarly, microglia isolated from the spinal cord of rats with SOD1^G93A^ mutation, a rodent model for amyotrophic lateral sclerosis (ALS), exhibited senescence with ~ 8 fold increases in SA-β gal staining by DIV12 indicating replicative senescence in microglia [41]. In addition to replicative senescence, DNA damage, mitochondrial dysfunction, oxidative stress, proteasome inhibition and beta-amyloid are some of the other common senescence inducing signals that accumulate with age and lead to premature senescence in all types of CNS glial cells [42]. *In vitro* studies in primary astrocyte cultures from rodents and humans show that astrocytes are more sensitive to oxidative stress and loss of proteasome function and acquire a senescence phenotype that includes decreased proliferation, increased senescence-associated β-galactosidase, and expression of senescence markers p16, p21, and p53 [43]. Also, oligodendrocyte progenitor cells undergo senescence in response to stress from exposure to amyloid β aggregates [25]. Although traditional wisdom excludes neuronal cells from undergoing senescence, some studies have challenged this notion [44]. Unfortunately, our experimental conditions did not yield viable neurons to investigate this in the current study but our future studies in p16 reporter mice will address whether or not senescence occurs in neurons with aging.

Glial cells play a supporting role in neuronal function through regulation of neurotransmission (receptor-mediated glutamate reuptake mechanisms), myelination, synaptogenesis, secretion of neurotrophic factors that promote neuronal survival, immune modulation and maintenance of the blood-brain barrier (BBB) [45]. Senescence can potentially affect the structural and functional characteristics of glial cells and in turn affect neuronal activity. We understand that our study is associative and does not explain the causal relationship between glial senescence in the brainstem and SNS dysregulation observed with aging. However, we would like to put forth several mechanisms that senescence-associated dysfunctional glia might engage to adversely affect the activity of pre-sympathetic neurons in the aged brainstem. First, senescence in astrocytes could affect the expression levels of glutamate reuptake transporter expression (excitatory amino acid transporter 1 and 2, EAAT1 and 2) and result in altered neurotransmission. Hydrogen peroxide (H202) induced senescence in rodent cortical astrocytes has been demonstrated to decrease glutamate uptake [46], which might lead to higher extracellular glutamate levels at the synapse and lead to sympathoexcitation. Secondly, the well-studied mechanism accounting for senescence-related pathologies is the SASP. In addition to promoting chronic inflammation, SASP secreted factors like proteases might alter the tissue microenvironment through degradation of the extracellular matrix and stimulate fibrosis [47]. They might also induce paracrine senescence in neighboring cells as a “bystander effect” and thereby propagate the senescence process. Lastly, even though senescent cells are resistant to apoptosis, they are still not exempt from immune surveillance by microglial cells and hence subject to clearance by phagocytosis. However, senescence in microglial cells impairs their ability in target detection, chemotaxis, migration and phagocytosis [48]. This will lead to prolonged aberrant persistence of senescent cells which can have detrimental effects in terms of chronic inflammation and sustained sympathoexcitation in aging.

One major limitation in this study is that our results are associative and did not address the causal relationship between senescence and sympathetic nerve overactivity in aging. Our future studies will address this using genetic and pharmacological means to eliminate senescent cells and investigate its effect on age-related sympathetic nerve overactivity. Despite increasing evidence that age-related SNS dysregulation is a huge risk factor for cardiovascular diseases, there are currently no therapeutic interventions to counteract this phenomenon. Our studies demonstrating an association between glial senescence in the brainstem and SNS activity in aging highlights the potential feasibility of senolytics for reducing age-associated cardiovascular risk, provided that future mechanistic studies validate this approach.

## MATERIALS AND METHODS

### Animals and treatment

Twenty-month-old male C57BL/6J mice were purchased from Jackson Laboratories (Bar Harbor, ME, USA). The animals were maintained on ad libitum food and water for 4 months. At the age of 24 months, the animals were sacrificed by cervical dislocation. Blood was collected, serum separated and stored at -80° C until further analysis. The brainstem was collected and either frozen on dry ice for RNA and protein analysis or stored in ice-cold PBS for neuron and glial cell isolation. For young controls, tissues were collected from 4-month-old C57BL/6J mice as in aged animals. All the animal protocols were approved by the Institutional Animal Care and Use Committee of the Oklahoma State University (IACUC number – VM-18-18) and were carried out under the guidelines of the National Institutes of Health Guide for the Care and Use of Laboratory Animals.

### Serum norepinephrine (NE) measurements

The levels of serum norepinephrine were measured with a commercial ELISA kit according to the manufacturer’s instructions (Labor Diagnostika Nord). Briefly, 15ul of serum was used for NE extraction from young and aged samples. Extracted supernatants were quantified using a competitive enzyme-linked immunoassay according to the instructions of the manufacturer. The final values after correction for the sample volume used for NE extraction were expressed in pg/ml units.

### Nuclear protein extraction

Nuclear protein was isolated from frozen brainstem tissue using the nuclear extract kit (Active Motif, CA) as per the manufacturer’s instructions. Protein concentrations in the nuclear isolates were estimated using MicroBCA assay (ThermoScientific).

### Measurement of NF-κB DNA-binding capacity by enzyme-linked immunosorbent assay (ELISA)

Equal amounts of nuclear protein (10μg) isolated from young and aged brainstem samples were assayed for their ability of nuclear NF-κB p65 to bind a DNA consensus sequence using an ELISA-based TransAM NF-κB p65 kit (Active Motif). The binding ability of nuclear NF-κB p65 was measured by color development and the absorbance was read at 450nm.

### Protein extraction and cytokine measurements

Frozen brainstem tissues from young and aged animals were homogenized in RIPA buffer with protease and phosphatase inhibitors, vortexed intermittently for 30 minutes in ice and centrifuged at 12000rpm for 10 minutes at 4C. The supernatant containing soluble protein was separated and the protein concentration was measured using Micro BCA protein assay (ThermoFisher Scientific). Cytokine and chemokine measurements from the protein extracts (25μl) were analyzed using Milliplex Mouse MAP cytokine/chemokine 32 plex kit and the values were normalized to protein concentration in the sample.

### RNA *in situ* hybridization (RNA-ISH)

RNA-ISH was performed using the chromogenic RNAscope 2.5 HD Assay – Red from Advanced Cell Diagnostics (ACD) on fresh frozen brain tissue sections as per the manufacturer’s instructions. Briefly, the brainstem was sectioned into 20μm thick sections. The slides were air-dried and then incubated in a dry air oven for 30 mins at 60° C and then washed with 1X PBS for 5 mins. The slides went through the manufacturer’s standard pre-treatment protocol. Following pre-treatment, the slides were incubated with mouse p16 probe (RNAscope Probe- Mm-Cdkn2a, Accession No:NM_009877.2) for 2h at 40° C, following which they were washed with wash buffer (WB) twice. Slides were then serially incubated with AMP1 (30min at 40° C), AMP2 (15min at 40° C), AMP3 (30min at 40° C), AMP4 (15min at 40° C), AMP5 (30min at RT), AMP6 (15min at RT) and were washed twice between each incubation. The slides were finally incubated with the RED working solution for 10min at RT and washed twice with water. The slides were counterstained with 50% Gill’s hematoxylin-I for 2 min at RT and 0.02% ammonia water was used as a bluing reagent. The slides were washed with water between each step and finally dried before mounting with Vectamount. We used custom-ordered sense probes from the same company (ACDbio) as a negative control to assure the specificity of the p16 signal (Supplementary Figure 1). For technical controls, we used Ppia (housekeeping gene-positive control) and DapB (bacterial gene, unrelated species, negative control) (Supplementary Figure 1). The slides were imaged and the cells positive for p16 expression were identified based on the presence of red foci. The number of positive cells were counted using the cell count plugin in the Image J Fiji software.

### Neuron and glial cell isolation from the freshly isolated brainstem

Isolation of neuron and glia enriched population of cells was performed using the Neuron isolation kit (Miltenyi Biotec) as per the manufacturer’s instructions. Isolated cells were collected in Trizol for RNA extraction and stored at -80° C until further analysis.

### RNA extraction and real-time PCR analysis

RNA was extracted from brainstem tissue samples and isolated cells from the brainstem using DirectZol RNA microprep kit (Zymo Research, Irvine, CA). The quantity and quality of RNA samples were assessed using a Nanodrop system and a high-Capacity cDNA Reverse Transcription Kit (Applied Biosystems) was subsequently used for cDNA synthesis. The primers used to amplify the SASP markers such as IL-1b, IL6, MCP1 and TNFα are described previously [28]. The following additional primers used were: p16 forward 5’- CGTACCCCGATTCAGGTGAT-3’ and reverse 5’-TTGAGCAGAAGAGCTGCTACGT-3’; p21 forward 5’- GACAAGAGG CCCAGTACTTC-3’ and reverse 5’- GCTTGGAGTGATAGAAATCTGTC-3’; p53 forward 5’- CACAGCGTGGTGGTACCTTA-3’ and reverse 5’- TCTTCTGTACGGCGGTCTCT-3’; CXCL1 forward 5’- ACCCGCTCGCTTCTCTGT-3’ and reverse 5’- AAGGGAGCTTCAGGGTCAAG-3’; MMP3 forward 5’-CAGACTTGTCCCGTTTCCAT-3’ and reverse 5’- GGTGCTGACTGCA TCAAAGA-3’; MMP13 forward 5’- AAGATGTGGAGTGCCTGATG-3’ and reverse 5’- AAGGCCTTCTCCACTTCAGA-3’; GFAP forward 5’- GAAGTTCGAGAACTCCGGGAG-3’ and reverse 5’-TTAGACCGATACCACTCCTCTG-3’; Iba1 forward 5’- GTCCTTGAAGCGAATGCTGG-3’ and reverse 5’-CATTCTCAAGATGGCAGATC-3’; Olig2 forward 5’- GCTGGCGCGAAACTACATC-3’ and reverse 5’-CGTAGATCTCGCTCACCAGT-3’; NeuN forward 5’- GTTAAAATGCCAGTCCCGTGG-3’ and reverse 5’-TCCTGATACACGACCCTGGA-3’;actin forward 5’- TGTCCACCTTCCAGCAGATGT and reverse 5’-AGCTCAGTAACAGTCCGCCTAG. Average Ct values of the gene targets analyzed by RT-PCR are listed in Supplementary Table 1). The mRNA fold change was calculated by the 2^ddct^ method and normalized against the housekeeping gene, actin.

### Senescence associated beta-galactosidase (SA-β-Gal) staining

SA-β-Gal staining was performed on coronal brainstem sections containing the RVLM region using the senescence β-galactosidase staining kit (Cat. No: 9860S) purchased from Cell Signaling Technology according to the manufacturer’s instructions. RVLM tissue sections (20 μm thick) from fixed brains collected from young and aged animals were allowed to air dry after removing them from -80° C freezer. Each slide was then rinsed with 1X phosphate-buffered saline (PBS). The slides were fixed with 1X formaldehyde for 5 minutes at room temperature. After fixing, the slides were washed with 1X PBS to remove the excess formaldehyde. The β-Galactosidase staining solution was prepared and adjusted to pH 6.0 as per the manufacturer’s protocol. The slides were then incubated in the β-Galactosidase staining solution at 37° C for 10-14 hours in a carbon dioxide (CO2) free incubator. The development of blue color was considered as the endpoint for the reaction. The number of positive cells was counted using the cell count plugin in the Image J Fiji software.

### Image acquisition and analysis

For both SA-β-gal and RNA-ISH, we stained brainstem sections with RVLM spanning from 7.32mm to 6.4mm caudal to Bregma from both young and aged animals. RVLM was identified under 10x magnification below the nucleus ambiguus (black box in Figure 1C, 1E) and zoomed in under 40x magnification for image acquisition. We counted the number of positively stained cells in the 40x images in which RVLM occupied the entire field and this way the count was normalized to the imaged area uniformly across all the animals. For each animal in both the groups, we stained two representative RVLM sections and imaged both sides. All four values (2 sections and 2 sides) were averaged to represent the count for each animal. All the images were obtained using the Olympus BX43 microscpe equipped with DP27 color camera system.

### Statistical analysis

All data are expressed as mean±SEM. All data are analyzed by student’s t-test. When F-test showed significant variances between the groups, Mann-Whitney non-parametric test was performed. A p-value of <0.05 was considered statistically significant.

## Supplementary Material

Supplementary Figure 1

Supplementary Table 1
