# Supplementary material for: Aging is associated with glial senescence in the brainstem - implications for age-related sympathetic overactivity
Source: Aging (Albany NY). 2021 May 26;13(10):13460–73. doi: 10.18632/aging.203111 (PMC8202881; doi:10.18632/aging.203111)
Supplement: Supplementary Figure 1 [file aging-13-203111-s001.pdf]

SUPPLEMENTARY FIGURE

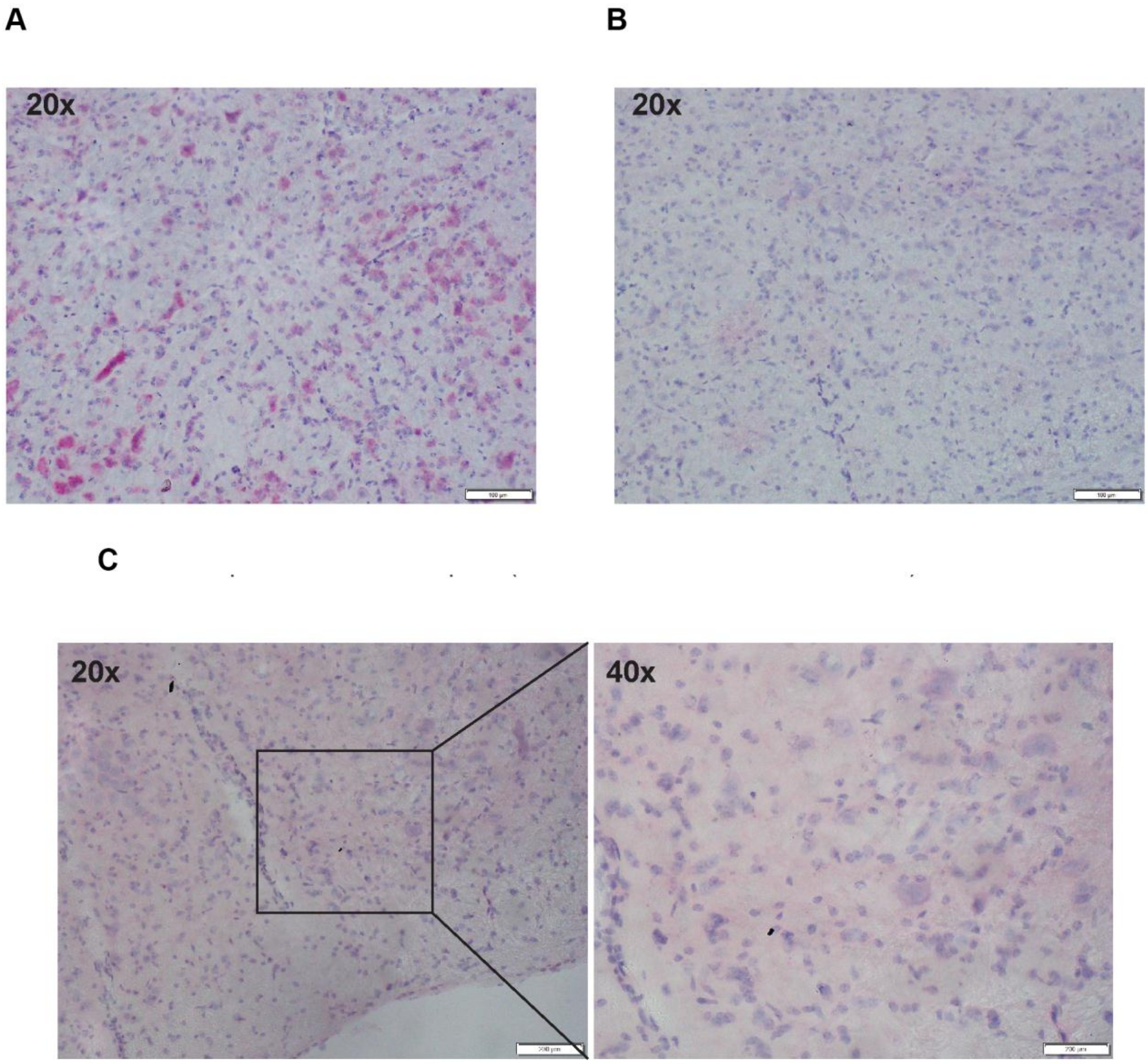

Supplementary Figure 1. Representative images for the positive and negative control probes used in the RNA-ISH experiment. (**A**) Positive Control probe Mm PPIB, House keeping gene (ACDBio, PN 313911). (**B**) Negative Control Probe dapB from bacteria Bacillus subtilis (ACDBio, PN 310043). (**C**) Sense probe to mouse p16 (custom ordered from ACDbio). Please note that control hybridization with sense probe gave no staining. Please note that we did not observe any staining (red foci) with the sense probes assuring the specificity of the p16 anti-sense probe used for our RNA-ISH experiments.
