# Supplementary material for: Aging is associated with glial senescence in the brainstem - implications for age-related sympathetic overactivity
Source: Aging (Albany NY). 2021 May 26;13(10):13460–73. doi: 10.18632/aging.203111 (PMC8202881; doi:10.18632/aging.203111)
Supplement: Supplementary Table 1 [file aging-13-203111-s002.pdf]

SUPPLEMENTARY TABLE

Supplementary Table 1. Average Ct values of the gene targets analyzed by RT-PCR.

Genes	Groups – Ct values obtained in RT-PCR analysis	
**Glia enriched fraction**	**Young**	**Aged**
p16	33.15	27
p21	24.29	24.03
p53	25.33	25.04
IL-1β	24.29	23.74
TNFα	25.03	23.6
MCP1	24.58	24.26
MMP3	26.35	25.69
**Brainstem tissue**		
p16	32.27	26.79
p21	20.77	21.10
p53	23.17	22.78
IL-1b	30.3	29.12
IL-6	31.42	31.29
IL-1a	29.73	27.79
MCP1	29.83	27.67
TNFα	29.75	28.26
MMP3	32.93	31.52
MMP13	29.4	29.51
